# Clinical and molecular analysis of a novel variant in heme oxygenase-1 deficiency: Unraveling its role in inflammation, heme metabolism, and pulmonary phenotype

**DOI:** 10.1016/j.ymgmr.2023.101038

**Published:** 2023-12-15

**Authors:** Lea-Sophie Berendes, Petra Schulze Westhoff, Helmut Wittkowski, Anja Seelhöfer, Georg Varga, Thorsten Marquardt, Julien H. Park

**Affiliations:** a University of Münster, Department of General Pediatrics, Münster, Germany; b University of Münster, Department of Pediatric Rheumatology and Immunology, Münster, Germany

**Keywords:** Heme oxygenase-1, Heme metabolism, Inflammation, Oxidative damage

## Abstract

Heme oxygenase 1 (HO-1) is the pivotal catalyst for the primary and rate-determining step in heme catabolism, playing a crucial role in mitigating heme-induced oxidative damage. Pathogenic variants in the *HMOX1* gene which encodes HO-1, are responsible for a severe, multisystem disease characterized by recurrent inflammatory episodes, organ failure, and an ultimately fatal course. Chronic hemolysis and abnormally low bilirubin levels are cardinal laboratory features of this disorder. In this study, we describe a patient with severe interstitial lung disease, frequent episodes of hyperinflammation non-responsive to immunosuppression, and fatal pulmonary hemorrhage. Employing exome sequencing, we identified two protein truncating variants in *HMOX1*, c.262_268delinsCC (p.Ala88Profs*51) and a previously unreported variant, c.55dupG (p.Glu19Glyfs*14). Functional analysis in patient-derived lymphoblastoid cells unveiled the complete absence of HO-1 protein expression and a marked reduction in cell viability upon exposure to hemin. These findings confirm the pathogenicity of the identified *HMOX1* variants, further underscoring their association with severe pulmonary manifestations . This study describes the profound clinical consequences stemming from disruptions in redox metabolism.

## 1 Introduction

Iron metabolism plays a pivotal role throughout human physiology, extending far beyond iron's fundamental function in oxygen transport. It serves as an essential cofactor for a myriad of enzymes involved in fundamental cellular processes, including DNA synthesis [1,2] and mitochondrial respiration [3]. Furthermore, iron's intrinsic association with redox homeostasis and signaling is underscored by its central role in the biosynthesis of heme yielding a prosthetic group found in hemoglobin, myoglobin, and various redox-active enzymes. Nevertheless, iron's utility is paradoxical, akin to a double-edged sword. While it is indispensable for catalyzing vital redox reactions, an excess of iron can trigger a cascade of events, culminating in oxidative stress and cellular damage [4,5]. Consequently, maintaining a delicate balance between iron acquisition, utilization, and sequestration is of paramount significance, as its perturbation can lead to iron overload, consequently triggering oxidative signaling pathways and exacerbating redox damage.

Heme oxygenases (HO) are pivotal enzymes converting heme into equimolar amounts of biliverdin, carbon monoxide (CO), and ferrous iron (Fe^2+^) [6]. In humans, two distinct isoforms exist: constitutively expressed heme oxygenase-2 (HO-2) and heme oxygenase-1 (HO-1), which is induced by various endogenous and exogenous stressors, mainly through a multitude of transcription factors [[7], [8], [9]].

The key role of HO-1 in heme catabolism as well as in response to increased oxidative stress is highlighted by the consequences of HO-1 deficiency in higher mammals [10,11]. Murine models of HO-1 deficiency demonstrated persistent inflammation and subsequent structural changes, indicating a dual role of HO-1 consisting of mitigation of oxidative damage and regulation of immune processes [12,13]. Human HO1-deficiency was first described by Yachie and colleagues in 1999 [14], and since then, ten additional cases have been documented (Table 1) [15]. The clinical presentation of these patients is diverse, ranging from a primarily inflammatory phenotype mimicking autoinflammatory disease to pulmonary manifestations, while a pathognomonic laboratory presentation with extreme hemolysis and low bilirubin levels could in theory facilitate early diagnosis based on simple laboratory parameters [15]. All affected individuals exhibit recurrent flares of hyperinflammation, oftentimes triggered by infections. Severe and ultimately fatal damage to various organs has been reported, primarily intracerebral hemorrhage [14,16] as well as severe interstitial lung disease [17]. Currently, no causative treatment options exist, although bone marrow transplant has been proposed as a potentially beneficial intervention based on results from murine models and a single case report [18,19].

**Table 1:** Overview of reported cases of HO-1 deficiency.

	Japanese patient (Yachie et al., 1999)	Indian patient (Radhakrishnan et al., 2011)	Indian patient (Radhakrishnan et al., 2011)	Indian patient (Gupta et al., 2015)	Turkish patient (Greil et al., 2016)	Indian patient (Yadav et al., 2018)	Iranian patient (Tahghighi et al., 2019)	US patient (Chau et al., 2020)	Indian Child (Renji et al., 2021)	Turkish patient (Dirim et al., 2023)	German patient (present manuscript)
Reference	J Clin Invest. 1999 Jan;103(1):129–35.	J Pediatr Hematol Oncol. 2011 Jan;33(1):74–8.	J Pediatr Hematol Oncol. 2011 Jan;33(1):74–8.	Scand J Rheumatol. 2016;45(2):165–6.	Haematologica. 2016 Nov;101(11):e436-e439.	Biol. Blood Marrow Transplant. 2018;24:S443.	Int J Mol Cell Med. 2019 Fall;8(4):300–307.	Pediatr Rheumatol Online J. 2020 Oct 16;18(1):80.	Indian Pediatr. 2021 Mar 15;58(3):290–291.	Clin Rheumatol. 2023 Feb;42(2):597–606.	
Sex	Male	Female	Male	Male	Male	Male	Female	Male	Male	Male	Male
*HMOX1* variant	c.24_144del; c.324_325del (p.R109X)	c.130C > T (p.R44X) homozygous	c.130C > T (p.R44X) homozygous	c.130C > T (p.R44X) homozygous	c.419G > T (p.G139V)	c.130C > T (p.R44X) homozygous	c.610 A > T (p.K204X) homozygous	c.262_268delGCCCTGGinsCC (p.Ala88Profs*51); c.636 + 2 T > A	homozygous nonsense variation in exon 3, no details reported	G139V homozygous	c.55dupG (p.Glu19Glyfs*14); c.262_268delinsCC (p.Ala88Profs*51)
Primary (organ) phenotype	vascular, renal	vascular, renal	N/A	N/A	Hemophagocytic lymphohistiocytosis (HLH)	N/A	N/A	pulmonary	N/A	renal, hepatic	pulmonary
Clinical presentation	recurrent fever, generalized erythematous rash, arthralgia	hemolysis, generalized inflammation, lymphadenopathy, nephritis	hemolysis, generalized inflammation, lymphadenopathy, nephritis	fever, hemolysis	microcytic anemia, progressive splenomegaly, hemophagocytic lymphohistiocytosis	fever, pallor, hypertension	fever, tachypnea, dyspnea, pericardial effusion, inflammation	chronic respiratory failure, hyperinflammatory flares	inflammation	AA-type renal amyloidosis due to chronic multisystemic inflammatory condition, chronic sclerosing osteomyelitis, liver cirrhosis, arthralgia	congenital heart disease, chronic inflammation, pneumonia
Age at first presentation	26 months	15 years	2 years	20 months	3 months	10 years	17 months	4 months	8 months	2 years	3 years
Growth retardation	+	−	+	−	N/A	short stature	−	+	+	N/A	+
Anemia	+	+	hemolysis	hemolysis	+	+	+	+	+	+	+
Leukocytosis	+	+	+	+	N/A	N/A	+	+	N/A	+	+
Thrombocytosis	+	+	+	+	N/A	+	+	+	thrombocytopenia	N/A	+
Hyperlipidemia	+	+	+	N/A	+	N/A	+	+	−	N/A	−
Coagulation abnormalities	+	+	+	N/A	+	N/A	+	+	−	N/A	+
Fever	+	+	+	+	+	+	+	+	+	+	+
Ferritin	elevated	elevated	elevated	elevated	elevated	elevated	elevated	elevated	elevated	elevated	elevated
Haptoglobin	elevated	elevated	N/A	elevated	normal	N/A	N/A	N/A	N/A	low	elevated
LDH	elevated	elevated	+	N/A	elevated	N/A	elevated	elevated	elevated	N/A	elevated
Spleen	asplenia	congenital asplenia	asplenia	small size	splenomegaly	asplenia	normal size	small size; later asplenia	normal size; later asplenia	splenomegaly; later splenic atrophy	splenomegaly; later normal size
Hepatomegaly	+	N/A	+	+	+	N/A	+	+	+	+	+
Proteinuria	+	+	+	+	N/A	+	N/A	´+	N/A	N/A	+
Treatment	oral steroids, erythrocyte transfusions	azithromycin, methylprednisolone, oral prednisolone, cyclophosphamide, rituximab	methylprednisolone, blood transfusions	N/A	immunochemotherapy (HLH protocol)	prednisolone, hydroxyurea, MMF, allogeneic stem cell transplantation	methylprednisolone, oral prednisolone, ibuprofen, cotrimoxazole	oral prednisolone, anakinra, cyclosporine, tocilizumab	oral steroids	intravenous antibiotics	intravenous and oral steroids
Cause of death	intracranial hemorrhage	intracranial hemorrhage, multiorgan failure, fungal sepsis	death following untreated fever and pallor	N/A	N/A	/	recurrent fever, hemorrhage, heart failure, ascites	respiratory failure	acute arterial stroke	unknown	fulminant pulmonary hemorrhage

In this article, we present a novel case of HO-1 deficiency, marked primarily by a lung-related phenotype with severe, recurring bouts of inflammation. Exome sequencing identified a novel protein truncating variant in *HMOX1* (c.55dupG (p.Glu19Glyfs*14)) on one allele and a known pathogenic protein truncation variant (c.262_268delinsCC (p.Ala88Profs*51)) on the other. Patient-derived lymphoblastoid cells were used in a functional assay of HO-1, thus demonstrating the pathogenicity of the combined variants.

## 2 Materials and methods

### 2.1 Study participants

Written informed consent of the participants or their legal guardians was obtained prior to inclusion in the study. The study was approved by the local ethics committee (Ethikkommission der Ärztekammer Westfalen-Lippe und der Westfälischen Wilhelms-Universität Münster, 2021–289-f-S).

### 2.2 Reagents and antibodies

All reagents were purchased from Sigma-Aldrich (St. Louis, MO, USA) unless stated otherwise. Antibodies as well as cell culture media and reagents were acquired from Thermo Fisher (Waltham, MA, USA). Flow cytometry materials including buffers and antibodies were purchased from Biolegend (San Diego, CA, USA).

### 2.3 Generation of patient-derived lymphoblastoid cells

EDTA blood samples collected from the patient, his mother, and two healthy controls. Blood was mixed with washing medium consisting of RPMI1640 (#11875093) with antibiotic-antimycotic (#15240062 according to the manufacturer's instruction), followed by centrifugation at 500*g* for 10 min. Immortalization was performed through transformation with supernatant of EBV-producing B95–8 marmoset B-lymphoblastoid cells to establish a lymphoblastoid cell line (LCL). LCLs were maintained in culture medium consisting of RPMI 1640, antibiotic-antimycotic, and 20% fetal bovine serum (#10270–106) at 37 °C and 5% CO_2_.

### 2.4 Genetic analysis

Trio exome sequencing of the blood from the patient and both parents as well as Sanger sequencing on blood and LCL samples was performed as previously described [20]. Identified variants were confirmed by conventional Sanger sequencing. Primer sequences and PCR conditions are available in the Supplementary Appendix.

### 2.5 Immunoblotting

Following induction of HO-1 by incubation of 1 × 10^7^ cells with 25 μM cadmium chloride (CdCl_2_) for 8 h at 37 °C and 5% CO_2_ [14,21], cells were lysed in RIPA buffer (150 mM NaCl, 1.0% IGEPAL® CA-630, 0.5% sodiumdesoxycholate, 0.1% SDS, 50 mM Tris, pH 8.0 + cOmplete protease inhibitor cocktail (Roche, Basel, Switzerland) according to the manufacturer's instructions) for 30 min on ice. An equal amount of non-treated cells was processed in an identical fashion. Supernatant was collected and diluted to a concentration of 1 μg protein/μL in RIPA buffer. After denaturation at 95 °C for 5 min, electrophoresis was performed on 15% polyacrylamide gels at 70 V for 30 min, followed by 105 min at 100 V. Proteins were transferred to PVDF membranes (Bio-Rad, Hercules, CA, USA, #1620175) using the Transblot Turbo system (Bio-Rad). Membranes were blocked in 5% (*w*/*v*) milk in TBST (20 mM Tris, 150 mM NaCl, 0.1% (w/v) Tween 20) at 4 °C overnight followed by incubation with an anti-human HO-1 antibody raised against amino acids 1–30 of the protein (# MA1–112, Invitrogen, Waltham, MA, USA, Fig. 2B) at a dilution of 1:1000 overnight at 4 °C. Equal loading was confirmed using an anti-human β-actin antibody (#sc-47,778,Santa Cruz Biotechnology, Dallas, TX, USA) at a dilution of 1:500 in TBST overnight at 4 °C. After three 10 min washes in phosphate buffered saline with Tween detergent (PBST, 137 mM NaCl, 2.7 mM, 10 mM Na_2_HPO_4_, 1.8 mM KH_2_PO_4_, 0.1% (*w*/*v*) Tween 20), membranes were incubated in HRP-conjugated secondary anti-mouse antibody (m-IgG κ BP-Hrp, Dako, Glostrup, Denmark) at a dilution of 1:2000. Cytiva AmershamECL Prime Western Blotting Detection Reagent (Cytiva, Marlborough, MA, USA) was used to detect the signal. Images were acquired using a Chemidoc imaging apparatus (Bio-Rad) and analyzed using ImageLab (Bio-Rad).

### 2.6 HO-1 functional assay

To assess the effect of a loss of HO-1 function, patient-derived (HMOX1 ^MUT/MUT^) and wild-type cells (HMOX1 ^WT/WT^) were exposed to porcine hemin (#51280) dissolved in NaOH at a final concentration of 1 mM [14]. Specifically, 2.5 × 10^5^ cells were incubated for 24 h at 37 °C in an atmosphere containin 5% CO_2_ with 75 μM hemin.

### 2.7 Flow cytometry and cell viability

Cells were washed with Dulbecco's phosphate buffered saline (# 14190144, DPBS) and Annexin V Binding Buffer (#422201)). followed by staining with 400 μl of Annexin V Binding Buffer, 4 μl of APC Annexin V (#640941, 1:100) and 4 μl of 7-AAD (#420404, 1:100). Cells were incubated in the dark on ice for 15 min. A total of 10,000 cells per sample were acquired and displayed in BD FACSDiva software. Data analysis was performed using the FlowJo software (version 10.8.0). Compensation for both antibodies was performed with single stained cells. Live and dead cells were distinguished by displaying their APC Annexin V and 7-AAD staining in a first step. Cells that were negative for both antibodies were analyzed regarding their forward scatter. Small cells were classified as cell debris and subtracted. Relative viability following hemin stimulation was calculated as follows:

$$\frac{\mathrm{percentage of viable cells following stimulation with hemin}}{\mathrm{baseline percentage of viable cells}}\times 100.$$

### 2.8 Statistical analyses

No assumption on normal distribution was made due to the small sample size. Means were compared using two-tailed Mann-Whitney *U* tests. Two-tailed *P* values <0.05 were considered to be statistically significant. Analyses were performed using GraphPad Prism (v10.0.2, GraphPad Software, Boston, MA, USA). Figures were created using biorender.com.

## 3 Results

### 3.1 HO-1 deficiency leads to a severe, inflammatory phenotype with interstitial lung disease and subsequently an ultimately fatal course

The patient, born to non-consanguineous parents, was delivered via Caesarean section following an uneventful pregnancy. At birth, the child's measurements were within normal ranges: body length (51.0 cm, 41st percentile), body weight (3600 g, 66th percentile), and head circumference (36.0 cm, 84th percentile). However, one month later, during a routine medical check-up, the patient was diagnosed with complex congenital heart disease with ventricular septal defect (VSD), atrial septal defect type II (ASD II), patent foramen ovale (PFO), narrow left ventricular outflow tract (LVOT), persistent left superior vena cava, mitral stenosis, and pulmonary hypertension. Upon further evaluation, hydrocephalus e vacuo was noted, and complete agenesis of the corpus callosum was suspected. Surgical corrections were performed when the patient reached ten months of age to address the VSD, narrow LVOT, aortic stenosis, and mitral stenosis. Postoperatively, the patient experienced an episode of high fever that did not respond to therapy but eventually resolved spontaneously.

**Fig. 1:**
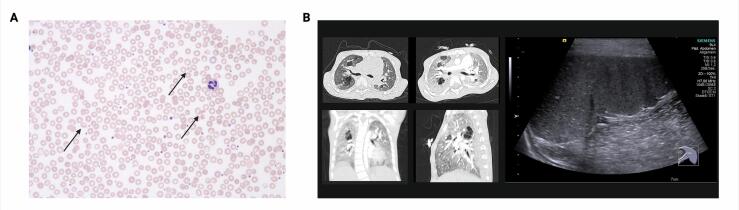
Clinical presentation of HO-1 deficiency. A. Mild changes of erythrocyte morphology were observed in peripheral blood smears. Fragmentocytes with helmet and crescent shapes are shown by arrows. B. Lung CT scans showed prominent structural changes, including bullae, bipulmonal peribronchiovascular thickening, and bipulmonary consolidations. In contrast to previously reported cases, splenomegaly was observed by ultrasound examination.

The patient was first seen at our Metabolic clinic at the age of 3 years and 2 months, and exhibited signs of growth retardation (body length: 94 cm, 16th percentile; body weight: 12.1 kg, 5th percentile; head circumference: 49.6 cm, 17th percentile). Additionally, the patient displayed facial dysmorphia, delayed gross motor skills development, muscular hypotonia, planovalgus feet, and pectus excavatum. Blood tests showed leukocytosis and elevated C-reactive protein (CRP) levels indicative of inflammation. Although there were signs of hemolysis (elevated lactate dehydrogenase, LDH), total bilirubin levels remained low. Erythrocyte morphology was mildly changed with the presence of fragmentocytes in helmet and crescent shapes (Fig. 1 A, arrows). Mild transaminase elevation suggested ongoing liver damage. Activated coagulation was evident from high d-dimer and fibrinogen levels. Urinalysis revealed elevated levels of taurine, cystine, phosphoethanolamine, and glycosaminoglycans (Supplementary Table 1). Sonography showed mild hepatosplenomegaly with a distorted spleen (Fig. 1 B). Genetic testing identified compound heterozygous nonsense mutations in *HMOX1*. Furthermore, array-based Comparative Genomic Hybridization (array-CGH) analysis revealed a 16p13.11 microduplication of paternal origin, which is known to potentially contribute to congenital heart disease, behavioral disorders, developmental delays, brain abnormalities, and skeletal malformations.

**Fig. 2:**
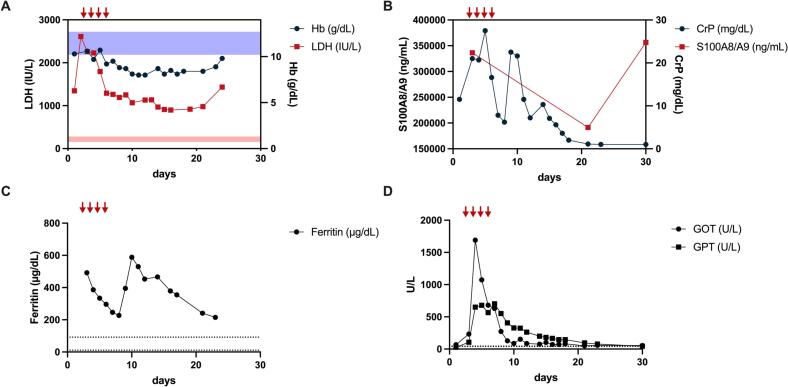
Hemolysis and inflammation are key features of HO-1 deficiency that are readily detected by conventional laboratory analyses. A. Chronic hemolytic anemia was identified in the patient and exacerbated upon an episode of viral infection as evidenced by low hemoglobin and elevated LDH levels. B + C.Conventional parameters of inflammation such as C-reactive protein (CrP), Ferritin, and S100A8/9 were highly elevated as well. D. Concurrently, liver failure with transaminase elevation was observed, while Steroid pulse therapy (red arrows) only had a temporary effect with a transient improvement of parameters followed by re-elevation.

At the age of 5, the patient was admitted for suspected pneumonia, presenting with fever, respiratory distress, cold symptoms, and crackling lung sounds. Blood tests indicated hyperinflammation with leukocytosis, and significantly elevated CRP, S100 A8/A9, ferritin, and procalcitonin levels, although no significant upregulation of interferon-regulated genes was observed in whole blood interferon signature analysis. Urinalysis showed proteinuria. During this episode, ultrasound revealed a regression of spleen size to normal. Treatment with dexamethasone pulse therapy led to temporary stabilization, with regression of fever and transiently improved inflammatory parameters (Fig. 2). Lung CT scans indicated prominent structural changes, including bullae, bipulmonal peribronchiovascular thickening, and bipulmonary consolidations (Fig. 1B). Oral administration of steroid therapy required high doses of prednisolone. The patient was evaluated for stem cell transplantation as a potentially curative intervention. However, he experienced sudden fulminant pulmonary bleeding and expired at the age of five years and eleven months.

### 3.2 c.55dupG (p.Glu19Glyfs*14) is a novel pathogenic variant in HMOX1

**Fig. 3:**
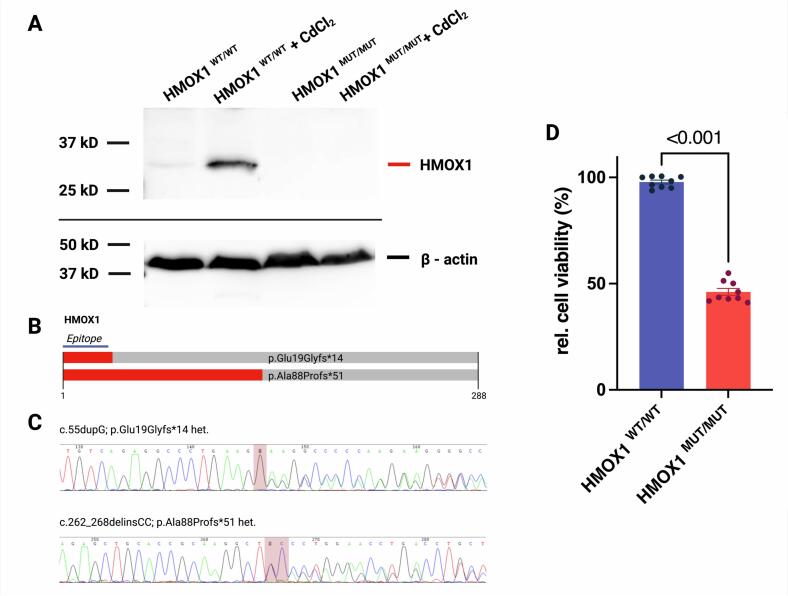
The truncating variants c.262_268delinsCC (p.Ala88Profs*51) and c.55dupG (p.Glu19Glyfs*14) in *HMOX1* cause HO-1 deficiency. A. Immunoblotting using an antibody against amino acids 1–30 of HO-1 readily detected the protein in control LCL upon stimulation with 25 μM CdCl_2_ for 8 h. In contrast, no protein expression was detected in patient-derived LCL. Shown is a representative blot of N = 3 replicates. B. Representation of structural consequences of the two detected truncating variants in *HMOX1*. The used antibody's epitope is indicated. C. Following identification in trio-exome sequencing, the protein-truncating variants c.262_268delinsCC (p.Ala88Profs*51) and c.55dupG (p.Glu19Glyfs*14) *in trans* were confirmed by conventional Sanger sequencing. Primer sequences are available in the

Trio exome sequencing identified the mutations c.55dupG (p.Glu19Glyfs*14); c.262_268delinsCC (p.Ala88Profs*51) *in trans* in a blood sample as well as in lymphoblasts (Fig. 3). c.262_268delinsCC (p.Ala88Profs*51) was inherited maternally and has been described previously [17]. The undescribed duplication variant c.55dupG (p.Glu19Glyfs*14) was inherited paternally. Both mutations result in a premature termination of the protein (Fig. 3 B). Immunoblotting confirmed the pathogenicity of both variants by demonstrating the total absence of HO-1 protein in patient-derived LCLs. While CdCl_2_ stimulation induced HO-1 expression in control cells, no such induction was observed in patient-derived LCLs. Control cells also showed slight increases HO-1 protein when left unstimulated (Fig. 3A). HO-2 was detected at identical levels by immunoblotting in both cell types, regardless of treatment (data not shown).

### 3.3 Loss of HO-1 leads to impaired cell viability upon hemin stimulation

Following stimulation of LCLs with 75 μM hemin, patient-derived LCLs (HMOX1 ^MUT/MUT^) showed a significant reduction in viability compared to controls (HMOX1 ^WT/WT^) (46.1 ± 4.9% vs 97.8 ± 2.6% respectively, *P* < 0.001), indicating absent HO-1 function and subsequent cell death (Fig. 3D).

## 4 Discussion

HO-1plays an indispensable role in maintaining cellular homeostasis and orchestrating multifaceted biological responses [22]. Consequently, disruption of heme degradation by variants in *HMOX1* may result in severe inborn errors of redox- as well as immuno-metabolism [14,[23], [24], [25]]. Although all previously reported pathogenic variants result in a hyperinflammatory phenotype with recurring bouts of primarily infection-triggered inflammation, but the principally affected organ systems vary among patients [15]. The involvement of the brain vasculature, kidneys, liver, and lungs might be attributed to location-specific immunity, but also due to the reported high levels of oxidative stress in these tissues [26].

Here, we present a case of a patient carrying a previously undescribed *HMOX1* variant and a known mutation. The c.55dupG (p.Glu19Glyfs*14) variant results in an early termination, leading to a total loss of protein function as evidenced by functional assessment with no protein detected in immunoblots, even upon stimulation with the known HO-1 inductor CdCl_2_ (Fig. 2 A). The predominantly pulmonary phenotype in conjunction with recurrent inflammatory episodes is in line with reports by Chau and colleagues and strengthens the link between a lack of HO-1 function and pulmonary inflammation [17]. In their report, the authors describe an equally severely affected individual with recurrent inflammatory episodes and interstitial lung disease with ultimately fatal course. Our patient's severe phenotype and a sudden infection-triggered onset following a pauci-symptomatic interval is comparable to previous reports that equally reported rapid deterioration following trigger episodes such as infections [23]. While some of the patient's phenotypical features have been described solely in association with the 16p13.11 microduplication (cardiac malformations, muscular hypotonia), others are shared by both conditions (failure to thrive / growth retardation) [27]. However, the defining phenotypical features – severe interstitial lung disease, frequent episodes of hyperinflammation non-responsive to immunosuppression, and fatal pulmonary hemorrhage – have been described in conjunction with HO-1 deficiency, albeit not in this particular combination [15].

There is strong evidence that inflammatory processes are the drivers of the phenotype in HO-1 deficiency [15]. Hemophagocytic lymphohistiocytosis (HLH)-like courses in addition to the known regulatory role of HO-1 on immune function [28,29] support the notion of immune dysregulation as a main pathomechanism underlying the condition. However, failed treatment attempts with various means of immunosuppression [17,24] such as steroid regimens as in our case hint at other mechanisms at least contributing to the pathogenesis. In our patient, massive elevations of certain general markers of inflammation (CRP, ferritin, S100A8/9 among others) were present whereas no significant change in the interferon signature (IFS) or proinflammatory interleukin levels could be observed. This might indicate a relevant contribution of downstream oxidative damage as a driver of the observed phenotype in addition to (redox-mediated) inflammation, given free heme's potent pro-oxidant effects [30]. HO-1 mice exhibit anemia, low serum iron levels, abnormal iron accumulation in the liver and kidneys, oxidative damage, and chronic, closely resembling the clinical presentations in patients [12,13]. Notably, the introduction of healthy macrophages can reverse these effects in HO-1 knockout mice, suggesting a potential usefulness of a similar approach in humans [31]. Indeed, allogenic stem cell transplantation has been suggested as a potential treatment and reported to lead to stabilization and prolonged survival in a single case [18]. However, given that oxidative damage is involved in the pathogenesis, anti-oxidant interventions might also have the potential to prevent downstream tissue damage and improve the disease course.

## 5 Conclusions

In conclusion, we have identified a novel *HMOX1* variant predominantly associated with pulmonary symptoms, particularly recurrent inflammation. This finding enriches our understanding of the varied clinical manifestations of this condition. It highlights the urgent necessity for developing innovative treatments, possibly leveraging antioxidant mechanisms.

## Funding

This research did not receive any specific grant from funding agencies in the public, commercial, or not-for-profit sectors.

## CRediT authorship contribution statement

**Lea-Sophie Berendes:** Data curation, Investigation, Methodology, Validation, Writing – original draft, Writing – review & editing. **Petra Schulze Westhoff:** Investigation, Methodology, Validation, Writing – review & editing. **Helmut Wittkowski:** Data curation, Investigation, Writing – review & editing. **Anja Seelhöfer:** Investigation, Validation, Writing – review & editing. **Georg Varga:** Data curation, Methodology, Validation, Writing – review & editing. **Thorsten Marquardt:** Investigation, Writing – review & editing. **Julien H. Park:** Conceptualization, Data curation, Formal analysis, Investigation, Methodology, Supervision, Validation, Visualization, Writing – original draft, Writing – review & editing.

## Declaration of Competing Interest

The authors have no conflicts of interest to disclose.

## Data Availability

The data supporting the results reported in this article, will be made available within 3 months from initial request to researchers who provide a methodologically sound proposal. The data will be provided after its de-identification in compliance with applicable privacy laws, data protection, and re-quirements for consent and anonymization.

## Appendix A Supplementary data

Supplementary material - Primer sequences Image 1

Supplementary Table 1 - Laboratory parameters of the patient Image 1
