# Supplementary material for: Clinical and molecular analysis of a novel variant in heme oxygenase-1 deficiency: Unraveling its role in inflammation, heme metabolism, and pulmonary phenotype
Source: Mol Genet Metab Rep. 2023 Dec 15;38:101038. doi: 10.1016/j.ymgmr.2023.101038 (PMC10764348; doi:10.1016/j.ymgmr.2023.101038)
Supplement: Supplementary file 1 — Supplementary material - Primer sequences [file mmc1.docx]

**SUPPLEMENTARY APPENDIX**

Clinical and Molecular Analysis of a Novel Variant in Heme Oxygenase-1 Deficiency: Unraveling Its Role in Inflammation, Heme Metabolism, and Pulmonary Presentation

Lea-Sophie Berendes ^1^, Petra Schulze Westhoff ^1^, Helmut Wittkowski ^2^, Anja Seelhöfer ^1^, Georg Varga ^2^, Thorsten Marquardt ^1^ & Julien H. Park ^1,*^

^1^ University of Münster, Department of General Pediatrics, Münster, Germany

^2^ University of Münster, Department of Pediatrics, Rheumatology and Immunology, Münster, Germany

* To whom correspondence should be addressed:
 Dr. med. Julien H. Park, University of Münster, Department of General Pediatrics, Albert-Schweitzer-Campus 1, Geb. A1, 48149 Münster, Germany. Email: [julien.park@ukmuenster.de](mailto:julien.park@ukmuenster.de)

1. Primer sequences

*HMOX1*

|  | Sequence | fragment length (bp) | annealing temperature (° C) |
| --- | --- | --- | --- |
| HMOX1_Ex1F(13U) | ATCAGCTGTTCCGCCTGGCCCAC | 356 | 66 |
| HMOX1_Ex1R(13R) | ACCGTTCCTCCCTCCAACTACCCT | 356 | 66 |
| HMOX1_Ex2F(13U) | AAGCGATTGAGAACGTGGCCTGAA | 511 | 65 |
| HMOX1_Ex2R(13R) | CAACATGGAACCGGTGCTCAGTCA | 511 | 65 |
| HMOX1_Ex3F(13U) | CTCTGGCTGCTGTGTGAAGAGGAT | 749 | 64 |
| HMOX1_Ex3R(13R) | CCATGACCTATAGCACCCTCCCCT | 749 | 64 |
| HMOX1_Ex4F(13U) | GTCCTACCTTCAGCTGGGACCTGG | 300 | 66 |
| HMOX1_Ex4R(13R) | GTGGGGCAGCAGGAAACAACACC | 300 | 66 |
| HMOX1_Ex5F(13U) | AGTTTAAGGAGAGGACAGGGAGCA | 773 | 62 |
| HMOX1_Ex5R(13R) | ACAAGGATACTTGAAACTCAGGGCTT | 773 | 62 |
| HMOX1_Ex5F2(13U) | CCTATGGCATCTTCCCCAACGAAA | 503 | 62 |
| HMOX1_Ex5R2(13R) | GGAGGAAAAGGTCAGTTCCCCAAG | 503 | 62 |
